# Sensory nerves have altered function contralateral to a monoarthritis and may contribute to the symmetrical spread of inflammation

**DOI:** 10.1111/j.1460-9568.2007.05737.x

**Published:** 2007-08

**Authors:** Sara Kelly, James Philip Dunham, Lucy Frances Donaldson

**Affiliations:** Department of Physiology and Pharmacology, School of Medical Sciences, University of Bristol Bristol, UK

**Keywords:** antidromic activity, arthritis, Complete Freund's Adjuvant, Evans blue, rat

## Abstract

Rheumatoid arthritis (RA) and rat models of RA exhibit symmetrical mirror-image spread. Many studies have sought to understand the underlying mechanisms and have reported contralateral effects that are manifested in many different forms. It is now well accepted that neurogenic mechanisms contribute to the symmetrical spread of inflammation. However, very few investigators have directly assessed changes in contralateral nerve function and there is a paucity of data. In the present study our aim was to investigate whether there are changes, in particular in the nervous system but also in the vascular system contralateral to an inflamed rat knee joint, that might precede overt inflammation and symmetrical spread. Three to five days following Complete Freund's Adjuvant (CFA) injection we found spontaneous antidromic (away from the CNS) activity in the homologous sensory nerve contralateral to the inflamed joint. Antidromic activity of this nature is known to result in the peripheral release of pro-inflammatory and vasoactive neuropeptides. Importantly, this activity was modulated by systemic analgesic treatment. Furthermore, levels of Evans blue dye extravasation were significantly increased in the joint contralateral to inflammation, indicating altered vascular function. These data suggest that contralateral increases in sensory neural activity and vascular function may account for the symmetrical spread of RA, and that early analgesic treatment may prevent or delay the spread of this debilitating disease.

## Introduction

Symmetry of inflammation is a fundamental characteristic of rheumatoid arthritis (RA; Arnett *et al*., 1988) and leads to widespread severe joint malformation and immobility (Clarke *et al*., 1994; Zangger *et al*., 2005). Despite a large literature on the aetiology and pathogenesis of RA, there is little understanding of the cause(s) of the symmetry of this disease.

Whilst the immune response is of obvious importance in RA progression, it is difficult to understand how this alone can underlie the symmetrical pattern of joint involvement. Levine and colleagues proposed that this symmetry might be underpinned by joint innervation, and proposed a neurogenic contribution to both existing inflammation and the symmetrical pattern of RA (Levine *et al*., 1985a,b; Levine & Basbaum, 1990). A considerable body of evidence now supports the ‘neurogenic’ hypothesis (Donaldson *et al*., 1995; Rees *et al*., 1996; Decaris *et al*., 1999). Epidemiological studies demonstrate that affected joints tend to show a mirror-image pattern (Egger *et al*., 1995; Zangger *et al*., 2005). Patients who develop RA after nerve injury show sparing of denervated joints (Thompson & Bywaters, 1962; Glick, 1967; Glynn & Clayton, 1976; Veale *et al*., 1993), and RA can resolve in affected joints that subsequently lose their sensory innervation (Lapadula *et al*., 1997).

Animal models of RA support the neurogenic hypothesis. The well-established Complete Freund's Adjuvant (CFA) model in rats shows symmetry of joint involvement after 2–4 weeks, depending on the site and concentration of CFA injection (Donaldson *et al*., 1993; Decaris *et al*., 1999). CFA-induced inflammation remains unilateral following lesioning of the contralateral homologous nerve with the neurotoxin capsaicin (Donaldson *et al*., 1995), implicating vanilloid receptor subtype 1 (TRPV1; also known as VR-1)-expressing neurons in the spread of inflammation. Further, mechanically evoked antidromic (away from the CNS) neuronal activity has been recorded in the contralateral articular nerve (Rees *et al*., 1996). The vasoactive neuropeptides substance P (SP) and calcitonin gene-related peptide (CGRP) are released from primary afferent terminals following activation (White & Helme, 1985); they increase both vascular permeability and vasodilatation (Brain *et al*., 1985; Ferrell & Russell, 1986; Holzer, 1998) and are chemotaxic for lymphocytes, thereby initiating inflammation (Helme *et al*., 1987; Mantyh, 1991; Holzer, 1998). Synovial SP and CGRP content increases rapidly and bilaterally following the induction of CFA arthritis (Bileviciute *et al*., 1993) and probably contributes to contralateral joint involvement (Lam *et al*., 2004).

Although it is now widely accepted that the nervous system plays an important role in the contralateral spread of inflammation, much of this understanding is based on nerve lesion studies. Very few studies have directly studied the function of contralateral nerves in models of RA. Our aim was to test the hypothesis that contralateral nerve function is altered following the development of arthritis. Further, our study aimed to determine whether and how this altered function might be modulated, and whether there are covert changes indicative of inflammation in the contralateral joint.

## Materials and methods

### Animals

All procedures on animals were performed in accordance with United Kingdom legislation [Animal (Scientific Procedures) Act, 1986], were licensed by the UK Home Office and follow the guidelines of the International Association for the Study of Pain (Zimmermann, 1983). Male Wistar rats (250–300 g) were obtained from B and K (Bristol, UK), housed four per cage and maintained on *ad libitum* food and water with a 12 h light–dark cycle.

### Drugs

Sodium pentobarbital was obtained in its sodium salt form from Sigma (Dorset, UK) and was made up in-house to allow i.p. and i.v. injection for anaesthetic induction and maintenance, respectively. Capsaicin was obtained from Tocris (Bristol, UK) and was made up in a 10% ethanol, 10% Tween 80 and 80% saline vehicle. Dipyrone (metamizol) was obtained from Sigma (Dorset, UK) and was dissolved in saline. Lignocaine, 2%, was obtained from Deproco Ltd (Kent, UK).

### Induction of CFA-induced inflammation

A brief anaesthesia was induced with halothane (3% in O_2_); once areflexia was achieved 250 µg/100 µL CFA (*Mycobacterium tuberculosis* in mineral oil vehicle) was injected through the patellar tendon into the right knee joint to induce arthritis (Rees *et al*., 1994). Rats were returned to the home cage and allowed to recover, and were monitored daily for gait disturbances. Ipsilateral and contralateral knee joint diameter measurements were taken from arthritic rats using callipers on the day of electrophysiological experiments.

### Surgical procedures

Three to five days following intra-articular injection of CFA, anaesthesia was induced with sodium pentobarbital (60 mg/kg, i.p.) and the external jugular vein and trachea were cannulated for maintenance of anaesthesia (10 mg/kg/ h, i.v.) and clearing of the airway, respectively. Core body temperature was maintained (36.5–37.5 °C) by the use of a rectal temperature probe connected to a heating lamp via a feedback control unit. The skin and connective tissues overlying the anterior aspects of the left knee and the medial aspect of the thigh were incised from the inguinal fossa to a point immediately below the medial condyle of the tibia. The lower part of the leg was attached to a platform for stability. The medial aspect of the leg was exposed and an oil pool was created using the skin of the hind-limb; this was filled with warmed (37 °C) mineral oil (Sigma, UK). The left saphenous nerve (contralateral to inflammation), which innervates the hind-limb including the knee joint, was dissected free from connective tissue at a point proximal to the knee joint and sectioned to prevent recording afferent activity from more distal parts of the rat hind-limb.

### Extracellular recording

The saphenous nerve was placed on a dental mirror, teased and placed onto bipolar platinum recording electrodes for differential recording of multiunit neuronal activity. Teased filaments were derived from the lateral aspect of the saphenous nerve, where knee joint afferents are located within the medial articular nerve as it runs parallel to and then joins the saphenous nerve. Identical recordings were also performed in control rats that did not receive an injection of CFA. In these preliminary experiments, neuronal activity was recorded for a 20-min period following placement of the nerve onto the electrodes. Recorded activity was amplified, filtered and captured for off-line analysis by a micro1401 (Cambridge Electronic Design, UK) and PC interface (Viglen) using Spike 2 software (Cambridge Electronic Design). Throughout, levels of activity were quantified as the frequency of action potentials (1-s bins) over 5-min periods. In some experiments, capsaicin (2%; *n* = 6) or lignocaine (2%; *n* = 6) was applied directly onto the nerve at a site proximal to the recording site, and effects on the frequency of the recorded activity were followed for between 20 and 25 min. In a separate set of experiments in CFA-injected rats (*n* = 12), following 1 h of baseline recordings of antidromic activity, dipyrone (50–250 mg/kg in saline) was injected i.v. and effects on neuronal activity were followed for 1 h per dose. The concentrations of dipyrone were selected on the basis of past work, demonstrating the inhibitory effect of dipyrone on spinal nociceptive transmission (Neugebauer *et al*., 1994). As vehicle controls, identical experiments were carried out except that dipyrone was replaced by four injections of saline (100 µL, i.v.) and levels of activity were followed for 1 h per administration of saline.

### Plasma extravasation experiments

Rats that had received a knee joint injection of CFA (4 days previously) were anaesthetized (pentobarbital 60 mg/kg, i.p.) and the external jugular vein and trachea cannulated. Following a period of time to allow for stabilization after the acute effects of surgery (∼30–40 min), Evans blue dye (50 mg/kg, i.v.) was injected. Twenty minutes after Evans blue administration rats were perfused transcardially with saline. Both knee joints were rapidly dissected, cleaned of skin and muscle, weighed and placed into formamide then onto a roller for 24 h at room temperature. Levels of extracted Evans blue dye were measured spectrophotomically (620 nm) and expressed as µg/mL/g. The Evans blue technique is a well-established method and has been routinely used to assess the effects of nociceptive afferent stimulation on vascular function (Janig & Lisney, 1989; Bharali & Lisney, 1992; Kolston & Lisney, 1993).

### Statistical analysis

Data are expressed as mean and SEM. Statistical analysis was carried out with nonparametric *t*-test (Mann–Whitney), paired *t*-test and nonparametric one-way anova, where appropriate.

## Results

### Development of arthritis

All rats that received an intra-articular injection of CFA, 3–5 days previously, developed a significant unilateral and localized swelling of the right knee joint (ipsilateral, 1.32 ± 0.03; contralateral, 0.91 ± 0.01 cm diameter; *P* < 0.0001, ipsilateral vs. contralateral, paired *t*-test, *n* = 32). In a separate behavioural study carried out using rats of the same sex, age and weight as those used in the present study, mean knee joint diameters (left and right) of control rats (*n* = 6) were 0.91 ± 0.01 cm (*P <* 0.001, Mann–Whitney). The ipsilateral knee joint diameters of CFA rats used in the present neurophysiological study were significantly swollen compared to these control knee joint diameters (*P <* 0.0001, Mann–Whitney), whereas contralateral knee joint diameters were identical.

Gait observations in CFA-injected rats confirmed that changes in nociceptive thresholds had occurred, as all rats favoured the injected hind-limb. We have previously noted that there is a significant decrease in the gait score and in the percentage of weight borne by the injected limb in this model (Kelly & Donaldson, 2005), in agreement with others' observations (Wilson *et al*., 2006).

### Recording of spontaneous antidromic activity in CFA rats

**Fig. 1:**
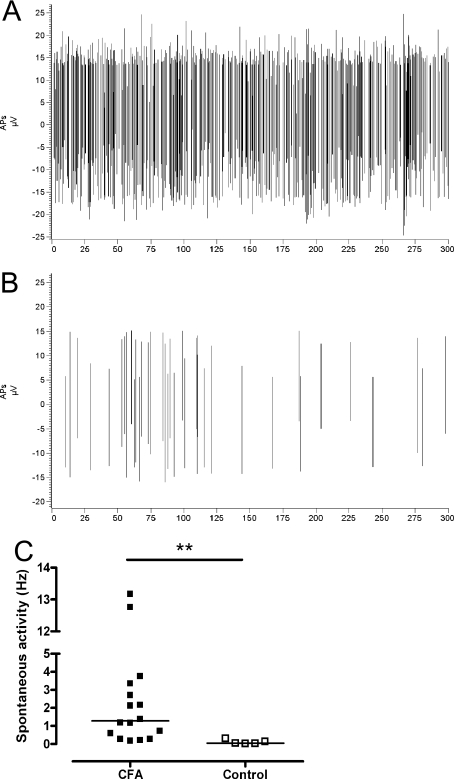
Effect of CFA-induced knee joint inflammation on contralateral antidromic activity. (A and B) Plot of spontaneous antidromic action potentials (peak to peak) recorded from the left saphenous nerve over a 5-min period from a typical experiment in (A) a CFA rat and (B) a control rat. (C) The mean frequency of spontaneous antidromic APs recorded from the left saphenous nerve over a 20-min period was significantly higher in rats that received an intra-articular injection of CFA (250 µg/100 µL; *n* = 16) into the right knee joint than in control noninjected rats (*n* = 5); **P < 0.01 vs. controls. Lines represent median levels of spontaneous activity.

Three to five days following the induction of CFA-induced inflammation of the right knee joint we were able to record both mechanically evoked (data not shown) and spontaneous antidromic neuronal activity from the left (contralateral) saphenous nerve (Fig. 1A and C). The mechanically evoked activity was neither robust nor reproducible and we therefore concentrated on the study of spontaneous neuronal activity. To determine whether this neuronal activity was due to the presence of inflammation in the right knee-joint, we carried out identical experiments in control noninjected rats (*n* = 5). Over the same time course of recording as in CFA rats (0–20 min), control rats exhibited very low levels of antidromic activity (Fig. 1B and C); when statistical comparisons were made between the two groups the activity recorded contralateral to inflammation in CFA rats was significantly higher than that seen in untreated control rats (*P <* 0.01, Mann–Whitney; Fig. 1C). In one experiment, sectioning of the saphenous nerve innervating the inflamed joint during recording, thereby interrupting enhanced input from the site of inflammation, had no effect on contralateral spontaneous activity (data not shown).

**Fig. 2:**
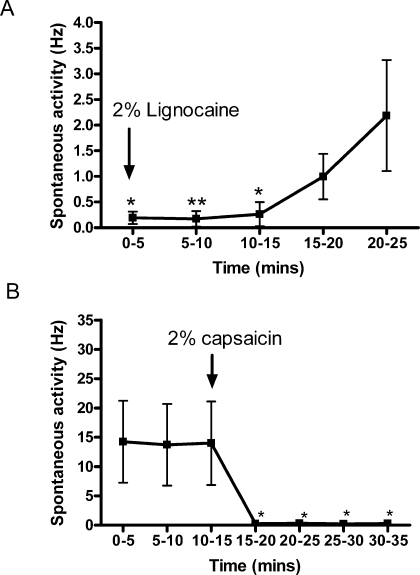
Spontaneous antidromic action potentials originated centrally and were transmitted by capsaicin-sensitive sensory neurons. (A) Lignocaine (2%) and (B) capsaicin (2%) applied directly onto the nerve proximal to the recording site in CFA rats (*n* = 5 or 6) blocked the generation of spontaneous antidromic APs. *P < 0.05, **P < 0.01.

In our experiments the saphenous nerve was sectioned distally to exclude the possibility that any recorded activity originated in the periphery rather than being centrally generated. To confirm that the recorded activity was truly of central origin we applied the local anaesthetic lignocaine (2%) directly onto the nerve proximal to the recording site (between the spinal cord and the contralateral joint; Fig. 2A). Lignocaine caused an almost complete abolition of the recorded activity within seconds of application (Fig. 2A). Spontaneous antidromic activity began to recover at ∼15–20 min and was complete by 1 h (data not shown). As the saphenous nerve is known to contain both sensory and sympathetic nerve fibres in an approximate ratio of 4 : 1 (Baron *et al*., 1995), we sought to clarify whether the activity observed was of sensory or sympathetic origin. We applied capsaicin (2%), an agonist at the TRPV1 receptor known to block sensory transmission, directly onto the nerve at a site proximal to the recording site (*n* = 6; Fig. 2B). Capsaicin was able to almost completely abolish the recorded activity, which did not recover over the time-course studied (Fig. 2B). In some experiments there was a small amount of residual activity present following application of capsaicin.

### Effects of systemic nonsteroidal anti-inflammatory drug administration on spontaneous antidromic activity in CFA rats

**Fig. 3:**
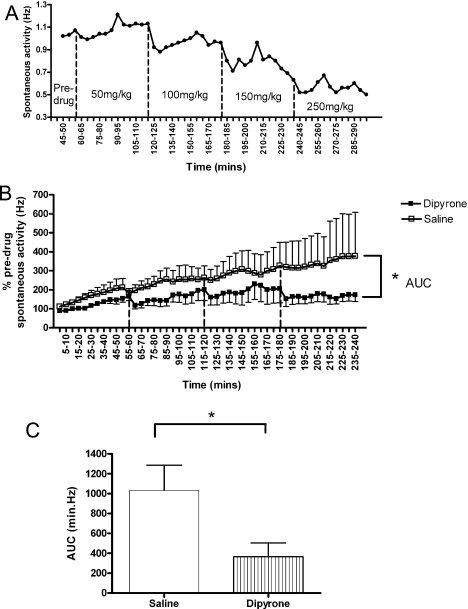
Effect of systemic dipyrone (50–250 mg/kg i.v.) on the frequency of spontaneous antidromic action potentials (APs). (A) An example of the time course of the effect of systemic dipyrone on the frequency of spontaneous antidromic APs in a single experiment. (B) Mean data for the effect of systemic dipyrone (50–250 mg/kg i.v.; *n* = 7) and saline (4 × 100 µL i.v.; *n* = 5) on the frequency of spontaneous antidromic APs in rats with CFA-induced inflammation of the right knee joint (*n* = 12). Dipyrone (50 mg/kg) or 100 µL saline administered at time 0; dotted lines represent the subsequent administration of dipyrone (100–250 mg/kg i.v.) or three further saline administrations (100 µL i.v. each). (C) Area under the curve (AUC) analysis for the cumulative effect of systemic dipyrone and saline on spontaneous antidromic APs; *P < 0.05.

In inflamed animals, in the absence of any drugs other than anaesthetic and saline (i.v.), the frequency of contralateral spontaneous activity increased over time (0–5 h; Fig. 3B). In order to investigate whether a clinically relevant pharmacological agent could modulate the increasing levels of spontaneous antidromic activity seen following CFA-induced inflammation, we administered the paracetamol (acetaminophen)-related centrally acting analgesic, dipyrone (50–250 mg/kg, i.v.), and studied the effect on the frequency of spontaneous antidromic action potentials. In most cases (5/7 experiments) dipyrone reduced the frequency of spontaneous antidromic activity to levels approaching or even below baseline levels of activity (Fig. 3A). Overall, the administration of dipyrone reduced the activity in contralateral sensory neurons, inhibiting the increase in activity over time that was apparent in saline control experiments (Fig. 3B). There was a significant effect of dipyrone over time, as shown by a significant reduction in the area under the curve (AUC) in the presence of dipyrone compared with saline control experiments (Fig. 3C; *P* < 0.05, Mann–Whitney).

### Plasma extravasation measurement in rat knee joints

When injected, intravascular Evans blue dye binds to plasma proteins and acts as a marker for areas of extravasation. We used this method to investigate whether there were differences in the level of plasma extravasation between joints that were contralateral to inflammation and showed no overt signs of inflammation, and those that were contralateral to normal noninflamed control joints.

**Fig. 4:**
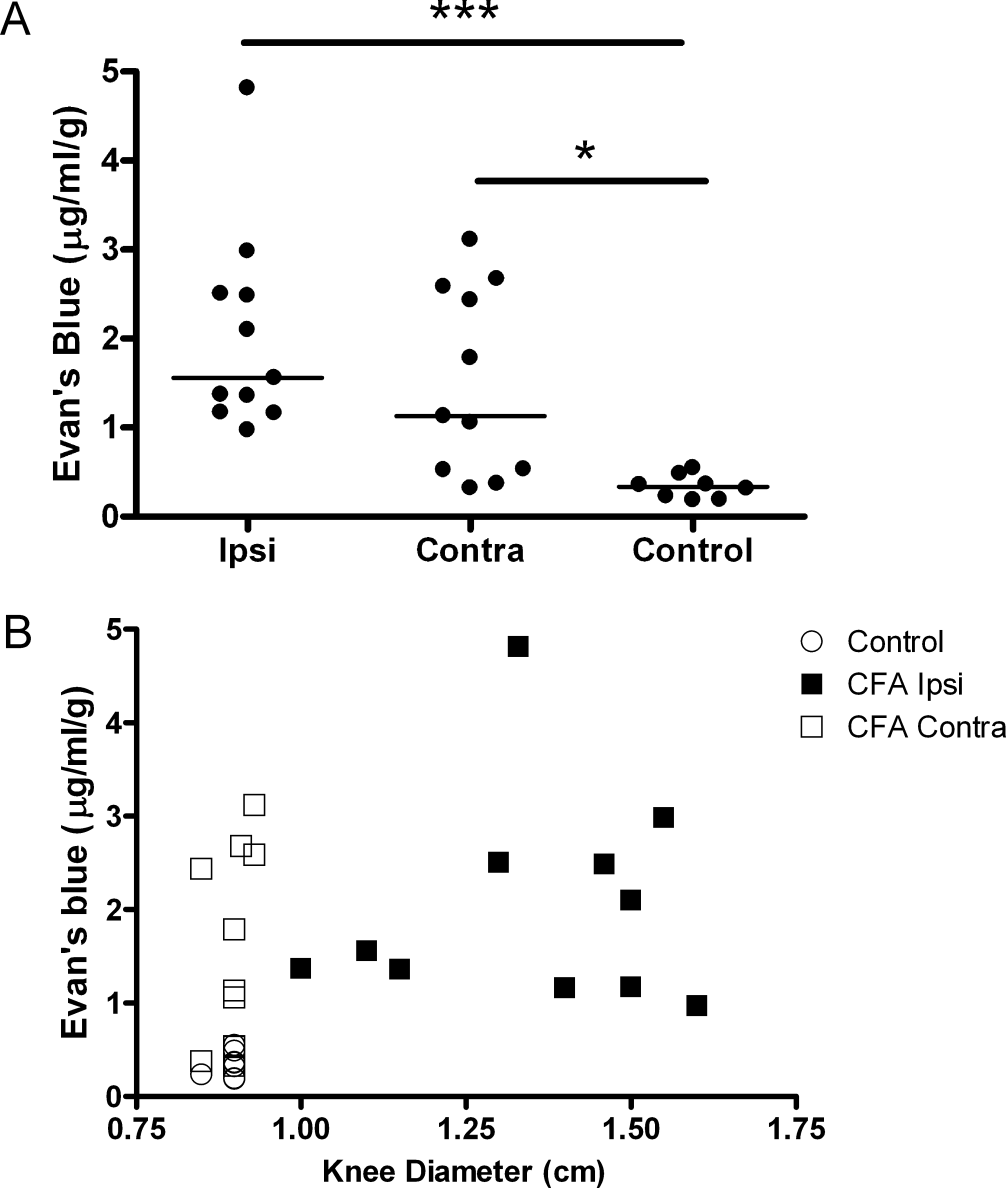
Effect of CFA-induced inflammation of the knee joint on ipsilateral and contralateral Evans blue dye extravasation (µg/mL/g tissue). (A) Amount of Evans blue extracted from the ipsilateral and contralateral knee joints of CFA rats (*n* = 11) was significantly greater than that extracted from knee joints (left and right) of control rats (*n* = 4), ***P < 0.001, *P < 0.05 vs. control. Lines represent medians. (B) Evans blue extravasation vs. ipsilateral (Ipsi), contralateral (Contra) and control (left and right) knee joint diameter.

When comparing the plasma extravasation seen in ipsilateral joints of CFA-inflamed rats and that seen in control noninflamed joints (right and left control joints were pooled as there were no significant differences between them), the level of extravasation was significantly greater, as expected (*P <* 0.001, Kruskal–Wallis). Importantly, the level of Evans blue extravasation seen in joints contralateral to inflammation was also significantly greater than that seen in control knee joints (Fig. 4A and B; *P* < 0.05, Kruskal–Wallis), and was not significantly different from the values for the inflamed joints. The median Evans blue extravasation in the contralateral joints was intermediate to the control and ipsilateral inflamed joints (Fig. 4A).

The Evans blue assay is frequently misinterpreted as a measure of vascular permeability (see Discussion) and might therefore be assumed to correlate well with oedema. However, when joint swelling (diameter) is compared to Evans blue extravasation, it is clear that although joints contralateral to inflammation have high Evans blue levels (Fig. 4B) they do not show any swelling. This indicates that Evans blue extravasation can be increased under circumstances where vascular permeability is not clearly altered. This implies that, in joints contralateral to an overt inflammation, vascular function is altered but vascular permeability (leading to oedema) probably is not.

## Discussion

Animal models of RA demonstrate symmetry of joint involvement (Donaldson *et al*., 1993), and provide an ideal opportunity for the study of underlying mechanisms and development of potential treatment strategies. In the past, use of these models, combined with nerve lesion, has provided evidence that suggests that neurogenic mechanisms are central to the development and maintenance of contralateral effects (Levine *et al*., 1985b; Bileviciute *et al*., 1993; Mapp *et al*., 1993; Donaldson *et al*., 1995; Kidd *et al*., 1995; Rees *et al*., 1996; Decaris *et al*., 1999; Lombard *et al*., 1999; Lam *et al*., 2004). However, few electrophysiological studies have been carried out to directly investigate the functional plasticity that occurs in contralateral sensory nerves following the induction of arthritis. Our data describe for the first time that a sensory nerve contralateral to arthritis conducts spontaneous action potentials towards the periphery. In addition, our data provide evidence that the function of the vascular system is also altered in the contralateral knee joint. We report that these changes occurred prior to overt signs of symmetrical inflammation and hypothesize that the reported plasticity has important implications for the spread of inflammation in this model and may mirror changes seen in man. Importantly, we were able to demonstrate that intervention with a centrally acting analgesic was able to modulate contralateral neuronal activity.

In the present study, contralateral spontaneous antidromic nerve activity was dependent on the presence of inflammation and could be abolished with lignocaine and capsaicin applied directly to the nerve trunk. Thus, we have confirmed that this activity was neural, originated centrally and occurred in TRPV1-expressing sensory fibres (Pini & Lynn, 1991). In some CFA animals we observed that a small amount of residual neuronal activity was present following capsaicin application, implying that there may be altered activity in contralateral sympathetic efferents (Helliwell *et al*., 1998; Tominaga *et al*., 1998; Smith *et al*., 2004; Moalem *et al*., 2005). However, perineural capsaicin prevents the symmetrical spread of arthritis in this model (Donaldson *et al*., 1995), implying that it is capsaicin-sensitive sensory neurons that are pivotal to the symmetrical progression of arthritis.

Antidromic activity in sensory nerves (dorsal root reflexes; DRRs) is a form of primary afferent depolarization (PAD) generated by the action of GABA at GABA_A_ receptors on the central terminals of primary afferents (Willis, 1999). Under physiological conditions PAD acts to reduce the amount of neurotransmitter released centrally leading to presynaptic inhibition (Willis, 1999). However, under pathological conditions DRRs may be generated and transmitted towards the periphery where they contribute to the development of inflammation (Rees *et al*., 1994; Lin *et al*., 1999). It is likely that the spontaneous antidromic neural activity we report represents spontaneous DRRs that could be an important contributor to the development of contralateral inflammation. Indeed, antidromic stimulation of sensory nerves leads to the release of the pro-inflammatory neuropeptides SP and CGRP (White & Helme, 1985). We have demonstrated that inflamed animals have greatly enhanced levels of spontaneous antidromic activity compared to noninflamed control animals, possibly due to the central sensitization of primary afferents. The increasing spontaneous activity we see over time in inflamed animals is probably a consequence of the interaction of the barbiturate anaesthetic pentobarbital with GABA_A_ receptors, further enhancing this already sensitized system (Nicoll & Wojtowicz, 1980; Feng *et al*., 2004). Mechanically evoked contralateral DRRs have previously been reported in this model, but are less robust and more inconsistent (Rees *et al*., 1996, and present study). Arthritic joints tend to be guarded and little weight is borne on them (Kelly & Donaldson, 2005; Wilson *et al*., 2006). As a consequence of this altered behaviour, mechanically evoked antidromic activity may contribute less to the neurogenic component involved in the development of contralateral inflammation than activity occurring spontaneously.

Here, we provide the first report of modulatory effects of a centrally acting analgesic on contralateral proinflammatory nerve activity. Systemic dipyrone, at concentrations that have inhibitory effects on spinal nociceptive transmission (Neugebauer *et al*., 1994), was able to inhibit spontaneous antidromic nerve activity. Inhibition was not, however, complete, suggesting that spontaneous contralateral DRRs may be well established only 3 days after the onset of unilateral inflammation. Although dipyrone inhibits activity in primary afferents innervating an arthritic joint (Neugebauer *et al*., 1994; Ferreira, 2002; Kelly & Donaldson, 2005), in a single experiment in which ipsilateral afferent input was interrupted there was no effect on spontaneous contralateral output, suggesting that a peripheral action of dipyrone may not be key in the effects we observe. Evidence suggests that both spinal and supraspinal pathways may be involved in the generation of contralateral responses (Thompson & Bywaters, 1962; Levine *et al*., 1985a; Bileviciute *et al*., 1998; Eide *et al*., 1999; Peng *et al*., 2001). Hence, it is likely that dipyrone acts at central sites to modulate contralateral responses (Beirith *et al*., 1998; Mazario & Herrero, 1999; Ferreira, 2002). Dipyrone has an inhibitory action on cyclooxygenase (COX) enzymes (Abbate *et al*., 1989; Abbate *et al*., 1990) and, in the periphery, stimulates the NO–cGMP pathway (Sachs *et al*., 2004); both of these proposed mechanisms may contribute to its reported analgesic action. COX2 mRNA and NO synthase are up-regulated bilaterally at the spinal cord level during peripheral inflammation (Beiche *et al*., 1996; Ichitani *et al*., 1997; Dolan *et al*., 2000). Importantly, prostaglandins (products of COX enzymes) and NO are involved in spinal nociceptive processing (Dirig *et al*., 1997; Tegeder *et al*., 2001; Vanegas & Schaible, 2001; Nakayama *et al*., 2004) and the regulation of the excitability of the central terminals of primary afferents (Beirith *et al*., 1998). It is likely, therefore, that the mechanism by which dipyrone modulated contralateral antidromic activity in the present study involves the inhibition of central COX and/or the stimulation of NO–cGMP activity.

Importantly, the spontaneous activity we report is of a frequency known to result in neurogenic inflammation following electrical stimulation of peripheral nerves, through release of SP and CGRP (White & Helme, 1985). SP and CGRP increase vascular permeability, perfusion (through vasodilatory effects) and plasma protein leakage from cutaneous vessels (Holzer, 1998), and are thought to play an important role in the contralateral spread of arthritis (Bileviciute *et al*., 1998; Decaris *et al*., 1999; Lam *et al*., 2004). In contralateral joints, there was an increase in plasma extravasation that was associated with the reported spontaneous antidromic sensory nerve activity but was not associated with overt swelling. The degree of Evans blue extravasation into tissue is a measure of combined effects on blood flow, capillary pressure, capillary permeability and many other factors (Bates *et al*., 1999). Many or all of these factors may be altered in early inflammation. Our findings indicate a change in one or several of these parameters in contralateral joints, but probably exclude a permeability change. Increased perfusion and vasodilation in the contralateral joints could be indicative of a ‘preinflammatory’ state that these data suggest occurs only 4 days after the onset of monoarthritis. These vascular changes are slightly smaller than those ipsilateral to the inflammation, a finding common to many studies (for reviews see Donaldson, 1999; Shenker *et al*., 2003).

Our findings indicate that central changes contributing to the generation of antidromic activity and the subsequent alterations in vascular function are already established by 3–5 days after CFA injection. The relatively short time frame of the reported changes is in agreement with previous reports of bilateral effects (Kidd *et al*., 1995; Rees *et al*., 1996). Interestingly, antidromic stimulation of nerves at C-fibre intensity evokes both unilateral (Janig & Lisney, 1989) and bilateral plasma extravasation (Ferrell & Russell, 1986), which suggests that altered sensory input to the spinal cord can induce very rapid changes in contralateral output. Knee joint plasma extravasation resulting from peripheral nerve stimulation is sensory afferent-dependent with no contribution from sympathetic efferents, which are, in fact, responsible for a diminution in plasma extravastion (Ferrell & Russell, 1986). Although sympathetic efferents have been implicated in the development of arthritis (Aloe *et al*., 1992; Bileviciute *et al*., 1995; Lubahn *et al*., 2004) and in the spread of inflammation (Levine *et al*., 1986) their possible contribution is still not clear.

To summarize, our data show that there is a neuronal correlate underlying contralateral inflammatory changes, manifested as ongoing antidromic activity in sensory neurons that is capable of altering contralateral articular perfusion very early in the disease. The early onset of contralateral changes will pose a challenge for the treatment of RA, but our finding that the centrally acting analgesic dipyrone can reduce the contralateral antidromic activity indicates that early intervention with this class of drugs may be useful in preventing the spread of arthritis. Understanding the neuronal mechanisms that underlie the spread of arthritis is of clear clinical relevance so that we may slow the progression of this destructive and debilitating disease.

## Abbreviations

CFA: Complete Freund's Adjuvant
CGRP: calcitonin gene-related peptide
DRR: dorsal root reflex
RA: rheumatoid arthritis
SP: substance P
TRPV1: vanilloid receptor subtype 1 (also known as VR-1).

## References

[b1] Abbate R, Gori AM, Pinto S, Attanasio M, Paniccia R, Coppo M, Castellani S, Giusti B, Boddi M, Neri Serneri GG (1990). Cyclooxygenase and lipoxygenase metabolite synthesis by polymorphonuclear neutrophils: in vitro effect of dipyrone. Prostaglandins Leukot. Essent. Fatty Acids.

[b2] Abbate R, Pinto S, Gori AM, Paniccia R, Coppo M, Neri Serneri GG (1989). Activity of dipyrone on intraplatelet arachidonic acid metabolism: an in vitro study. Pharmacol. Res..

[b3] Aloe L, Tuveri MA, Levi-Montalcini R (1992). Studies on carrageenan-induced arthritis in adult rats: presence of nerve growth factor and role of sympathetic innervation. Rheumatol. Int..

[b4] Arnett FC, Edworthy SM, Bloch DA, McShane DJ, Fries JF, Cooper NS, Healey LA, Kaplan SR, Liang MH, Luthra HS, Medsger TA, Mitchell DM, Neustadt DH, Pinals RS, Schaller JG, Sharp JT, Wilder RL, Hunder GG (1988). The American Rheumatism Association 1987 revised criteria for the classification of rheumatoid arthritis. Arthritis Rheum.

[b5] Baron R, Janig W, With H (1995). Sympathetic and afferent neurones projecting into forelimb and trunk nerves and the anatomical organization of the thoracic sympathetic outflow of the rat. J. Auton. Nerv. Syst..

[b6] Bates DO, Lodwick D, Williams B (1999). Vascular endothelial growth factor and microvascular permeability. Microcirculation.

[b7] Beiche F, Scheuerer S, Brune K, Geisslinger G, Goppelt-Struebe M (1996). Up-regulation of cyclooxygenase-2 mRNA in the rat spinal cord following peripheral inflammation. FEBS Lett..

[b8] Beirith A, Santos AR, Rodrigues AL, Creczynski-Pasa TB, Calixto JB (1998). Spinal and supraspinal antinociceptive action of dipyrone in formalin, capsaicin and glutamate tests. Study of the mechanism of action. Eur. J. Pharmacol..

[b9] Bharali LA, Lisney SJ (1992). The relationship between unmyelinated afferent type and neurogenic plasma extravasation in normal and reinnervated rat skin. Neuroscience.

[b10] Bileviciute I, Lundeberg T, Ekblom A, Theodorsson E (1993). Bilateral changes of substance P-, neurokinin A-, calcitonin gene-related peptide- and neuropeptide Y-like immunoreactivity in rat knee joint synovial fluid during acute monoarthritis. Neurosci. Lett..

[b11] Bileviciute I, Stenfors C, Theodorsson E, Beckman M, Lundeberg T (1995). Significant changes in neuropeptide concentrations in the brain of normotensive (WKY) and spontaneously hypertensive (SHR) rats following knee joint monoarthritis. Brain Res..

[b12] Bileviciute I, Stenfors C, Theodorsson E, Lundeberg T (1998). Unilateral injection of calcitonin gene-related peptide (CGRP) induces bilateral oedema formation and release of CGRP-like immunoreactivity in the rat hindpaw. Br. J. Pharmacol..

[b13] Brain SD, Williams TJ, Tippins JR, Morris HR, MacIntyre I (1985). Calcitonin gene-related peptide is a potent vasodilator. Nature.

[b14] Clarke GS, Buckland-Wright JC, Grahame R (1994). Symmetry of radiological features in the wrist and hands of patients with early to moderate rheumatoid arthritis: a quantitative microfocal radiographic study. Br. J. Rheumatol..

[b15] Decaris E, Guingamp C, Chat M, Philippe L, Grillasca JP, Abid A, Minn A, Gillet P, Netter P, Terlain B (1999). Evidence for neurogenic transmission inducing degenerative cartilage damage distant from local inflammation. Arthritis Rheum.

[b16] Dirig DM, Konin GP, Isakson PC, Yaksh TL (1997). Effect of spinal cyclooxygenase inhibitors in rat using the formalin test and in vitro prostaglandin E2 release. Eur. J. Pharmacol..

[b17] Dolan S, Field LC, Nolan AM (2000). The role of nitric oxide and prostaglandin signaling pathways in spinal nociceptive processing in chronic inflammation. Pain.

[b18] Donaldson LF (1999). Unilateral arthritis: contralateral effects. Trends Neurosci..

[b19] Donaldson LF, McQueen DS, Seckl JR (1995). Neuropeptide gene expression and capsaicin-sensitive primary afferents: maintenance and spread of adjuvant arthritis in the rat. J. Physiol. (Lond.).

[b20] Donaldson LF, Seckl JR, McQueen DS (1993). A discrete adjuvant-induced monoarthritis in the rat: effects of adjuvant dose. J. Neurosci. Meth..

[b21] Egger P, Cooper C, Hart DJ, Doyle DV, Coggon D, Spector TD (1995). Patterns of joint involvement in osteoarthritis of the hand: the Chingford Study. J. Rheumatol..

[b22] Eide AL, Glover J, Kjaerulff O, Kiehn O (1999). Characterization of commissural interneurons in the lumbar region of the neonatal rat spinal cord. J. Comp. Neurol..

[b23] Feng HJ, Bianchi MT, Macdonald RL (2004). Pentobarbital differentially modulates alpha1beta3delta and alpha1beta3gamma2L GABAA receptor currents. Mol. Pharmacol..

[b24] Ferreira SH (2002). Peripheral analgesic sites of action of anti-inflammatory drugs. Int. J. Clin. Pract. Suppl..

[b25] Ferrell WR, Russell NJ (1986). Extravasation in the knee induced by antidromic stimulation of articular C fibre afferents of the anaesthetized cat. J. Physiol. (Lond.).

[b26] Glick EN (1967). Asymmetrical rheumatoid arthritis after poliomyelitis. Br. Med. J..

[b27] Glynn JJ, Clayton ML (1976). Sparing effect of hemiplegia on tophaceous gout. Ann. Rheum Dis.

[b28] Helliwell RJ, McLatchie LM, Clarke M, Winter J, Bevan S, McIntyre P (1998). Capsaicin sensitivity is associated with the expression of the vanilloid (capsaicin) receptor (VR1) mRNA in adult rat sensory ganglia. Neurosci. Lett..

[b29] Helme RD, Eglezos A, Hosking CS (1987). Substance P induces chemotaxis of neutrophils in normal and capsaicin-treated rats. Immunol. Cell Biol..

[b30] Holzer P (1998). Neurogenic vasodilatation and plasma leakage in the skin. Gen. Pharmacol..

[b31] Ichitani Y, Shi T, Haeggstrom JZ, Samuelsson B, Hokfelt T (1997). Increased levels of cyclooxygenase-2 mRNA in the rat spinal cord after peripheral inflammation: an in situ hybridization study. Neuroreport.

[b32] Janig W, Lisney SJ (1989). Small diameter myelinated afferents produce vasodilatation but not plasma extravasation in rat skin. J. Physiol. (Lond.).

[b33] Kelly S, Donaldson LF (2005). Evidence for a role of peripheral prostaglandins in determining the mechanical sensitivity of primary afferent C-fibres of the rat knee joint. J. Physiol. (Lond.).

[b34] Kidd BL, Cruwys SC, Garrett NE, Mapp PI, Jolliffe VA, Blake DR (1995). Neurogenic influences on contralateral responses during experimental rat monoarthritis. Brain Res..

[b35] Kolston J, Lisney SJ (1993). A study of vasodilator responses evoked by antidromic stimulation of A delta afferent nerve fibers supplying normal and reinnervated rat skin. Microvasc. Res..

[b36] Lam FF, Wong HH, Ng ES (2004). Time course and substance P effects on the vascular and morphological changes in adjuvant-induced monoarthritic rats. Int. Immunopharmacol..

[b37] Lapadula G, Iannone F, Zuccaro C, Covelli M, Grattagliano V, Pipitone V (1997). Recovery of erosive rheumatoid arthritis after human immunodeficiency virus-1 infection and hemiplegia. J. Rheumatol..

[b38] Levine JD, Basbaum AI (1990). Neurogenic mechanism for symmetrical arthritis. Lancet.

[b39] Levine J, Collier D, Basbaum A, Moskowitz M, Helms C (1985a). Hypothesis: The nervous system may contribute to the pathophysioogy of rheumatoid arthritis. J. Rheumatol..

[b40] Levine JD, Dardick SJ, Basbaum AI, Scipio E (1985b). Reflex neurogenic inflammation. I. Contribution of the peripheral nervous system to spatially remote inflammatory responses that follow injury. J. Neurosci..

[b41] Levine JD, Dardick SJ, Roizen MF, Helms C, Basbaum AI (1986). Contribution of sensory afferents and sympathetic efferents to joint injury in experimental arthritis. J. Neurosci..

[b42] Lin Q, Wu J, Willis WD (1999). Dorsal root reflexes and cutaneous neurogenic inflammation after intradermal injection of capsaicin in rats. J. Neurophysiol..

[b43] Lombard MC, Weil-Fugazza J, Ries C, Allard M (1999). Unilateral joint inflammation induces bilateral and time-dependent changes in neuropeptide FF binding in the superficial dorsal horn of the rat spinal cord: implication of supraspinal descending systems. Brain Res..

[b44] Lubahn CL, Schaller JA, Bellinger DL, Sweeney S, Lorton D (2004). The importance of timing of adrenergic drug delivery in relation to the induction and onset of adjuvant-induced arthritis. Brain Behav. Immun..

[b45] Mantyh PW (1991). Substance P and the inflammatory and immune response. Ann. NY Acad. Sci..

[b46] Mapp PI, Terenghi G, Walsh DA, Chen ST, Cruwys SC, Garrett N, Kidd BL, Polak JM, Blake DR (1993). Monoarthritis in the rat knee induces bilateral and time-dependent changes in substance P and calcitonin gene-related peptide immunoreactivity in the spinal cord. Neuroscience.

[b47] Mazario J, Herrero JF (1999). Antinociceptive effects of metamizol (dipyrone) in rat single motor units. Neurosci. Lett..

[b48] Moalem G, Grafe P, Tracey DJ (2005). Chemical mediators enhance the excitability of unmyelinated sensory axons in normal and injured peripheral nerve of the rat. Neuroscience.

[b49] Nakayama Y, Omote K, Kawamata T, Namiki A (2004). Role of prostaglandin receptor subtype EP1 in prostaglandin E2-induced nociceptive transmission in the rat spinal dorsal horn. Brain Res..

[b50] Neugebauer V, Schaible HG, He X, Lucke T, Gundling P, Schmidt RF (1994). Electrophysiological evidence for a spinal antinociceptive action of dipyrone. Agents Actions.

[b51] Nicoll RA, Wojtowicz JM (1980). The effects of pentobarbital and related compounds on frog motoneurons. Brain Res..

[b52] Peng YB, Wu J, Willis WD, Kenshalo DR (2001). GABA (A) and 5-HT (3) receptors are involved in dorsal root reflexes: possible role in periaqueductal gray descending inhibition. J. Neurophysiol..

[b53] Pini A, Lynn B (1991). C-fibre Function During the 6 Weeks Following Brief Application of Capsaicin to a Cutaneous Nerve in the Rat. Eur. J. Neurosci..

[b54] Rees H, Sluka KA, Lu Y, Westlund KN, Willis WD (1996). Dorsal root reflexes in articular afferents occur bilaterally in a chronic model of arthritis in rats. J. Neurophysiol..

[b55] Rees H, Sluka KA, Westlund KN, Willis WD (1994). Do dorsal root reflexes augment peripheral inflammation?. Neuroreport.

[b56] Sachs D, Cunha FQ, Ferreira SH (2004). Peripheral analgesic blockade of hypernociception: activation of arginine/NO/cGMP/protein kinase G/ATP-sensitive K+ channel pathway. Proc. Natl Acad. Sci. USA.

[b57] Shenker N, Haigh R, Roberts E, Mapp P, Harris N, Blake D (2003). A review of contralateral responses to a unilateral inflammatory lesion. Rheumatology (Oxford).

[b58] Smith MP, Beacham D, Ensor E, Koltzenburg M (2004). Cold-sensitive, menthol-insensitive neurons in the murine sympathetic nervous system. Neuroreport.

[b59] Tegeder I, Niederberger E, Vetter G, Brautigam L, Geisslinger G (2001). Effects of selective COX-1 and – 2 inhibition on formalin-evoked nociceptive behaviour and prostaglandin E (2) release in the spinal cord. J. Neurochem..

[b60] Thompson M, Bywaters EG (1962). Unilateral rheumatoid arthritis following hemiplegia. Ann. Rheum. Dis.

[b61] Tominaga M, Caterina MJ, Malmberg AB, Rosen TA, Gilbert H, Skinner K, Raumann BE, Basbaum AI, Julius D (1998). The cloned capsaicin receptor integrates multiple pain-producing stimuli. Neuron.

[b62] Vanegas H, Schaible HG (2001). Prostaglandins and cyclooxygenases [correction of cycloxygenases] in the spinal cord. Prog. Neurobiol..

[b63] Veale D, Farrell M, Fitzgerald O (1993). Mechanism of joint sparing in a patient with unilateral psoriatic arthritis and a longstanding hemiplegia. Br. J. Rheumatol..

[b64] White DM, Helme RD (1985). Release of substance P from peripheral nerve terminals following electrical stimulation of the sciatic nerve. Brain Res..

[b65] Willis WD (1999). Dorsal root potentials and dorsal root reflexes: a double-edged sword. Exp. Brain Res..

[b66] Wilson AW, Medhurst SJ, Dixon CI, Bontoft NC, Winyard LA, Brackenborough KT, De Alba J, Clarke CJ, Gunthorpe MJ, Hicks GA, Bountra C, McQueen DS, Chessell IP (2006). An animal model of chronic inflammatory pain: pharmacological and temporal differentiation from acute models. Eur. J. Pain.

[b67] Zangger P, Keystone EC, Bogoch ER (2005). Asymmetry of small joint involvement in rheumatoid arthritis: prevalence and tendency towards symmetry over time. Joint Bone Spine.

[b68] Zimmermann M (1983). Ethical guidelines for investigations of experimental pain in conscious animals. Pain.

